# Effect of omega‐3 and vitamin D co‐supplementation on psychological distress in reproductive‐aged women with pre‐diabetes and hypovitaminosis D: A randomized controlled trial

**DOI:** 10.1002/brb3.2342

**Published:** 2021-09-02

**Authors:** Masoumeh Rajabi‐Naeeni, Mahrokh Dolatian, Mostafa Qorbani, Amir Abbas Vaezi

**Affiliations:** ^1^ Department of Midwifery and Reproductive Health, Student Research Committee, School of Nursing and Midwifery Shahid Beheshti University of Medical Sciences Tehran Iran; ^2^ Midwifery and Reproductive Health Research Center, Department of Midwifery and Reproductive Health, School of Nursing and Midwifery Shahid Beheshti University of Medical Sciences Tehran Iran; ^3^ Non‐communicable Diseases Research Center Alborz University of Medical Sciences Karaj Iran; ^4^ Chronic Diseases Research Center, Endocrinology and Metabolism Population Sciences Institute Tehran University of Medical Sciences Tehran Iran; ^5^ Department of Internal Medicine School of Medicine Alborz University of Medical Sciences Karaj Iran

**Keywords:** anxiety, depression, omega‐3 fatty acids, prediabetic state, vitamin D

## Abstract

**Purpose:**

Psychological distresses and pre‐diabetes are among the risk factors of developing type‐II diabetes. The present study was conducted to determine the effectiveness of omega‐3 and vitamin D co‐supplementation on psychological distresses in women of reproductive age with pre‐diabetes and hypovitaminosis D.

**Methods:**

The present factorial clinical trial was conducted on 168 women of reproductive age with pre‐diabetes and hypovitaminosis D. These participants were selected by stratified random sampling and were assigned to four groups for 8 weeks: group 1 (placebo group), group 2 (omega‐3 group), group 3 (vitamin D group), and group 4 (co‐supplement group). The medication and placebo doses being two 1000‐mg tablets each day for omega‐3 and 50,000‐IU pearls every 2 weeks for vitamin D. Fasting blood glucose and vitamin D were measured at the beginning of the study. The Depression Anxiety Stress Scale‐21 and the Pittsburgh Sleep Quality Index were completed by the participants at the start and end of the intervention.

**Results:**

A significant difference was observed in terms of reduction in anxiety and improvement in sleep quality in the co‐supplementation compared to the other three groups (*p* < .05). There was also a significant difference between the group receiving both supplements and the group receiving only placebos in terms of reduction in depression and stress (*p* < .05).

**Conclusion:**

Vitamin D and omega‐3 co‐supplementation improved depression, anxiety, and sleep quality in women of reproductive age with pre‐diabetes and hypovitaminosis D. Therefore, these two supplements can be recommended for improving the mental health of this group of women.

**Clinical trial registry:**

Iranian Registry of Clinical Trials Code: IRCT20100130003226N17. Registered on February 9, 2019.

## Abstract 2

Psychological distresses and pre‐diabetes are among the risk factors of developing type‐II diabetes. This study was conducted to determine the effectiveness of omega‐3 and vitamin D co‐supplementation on psychological distresses in women of reproductive age with pre‐diabetes and hypovitaminosis D.

## 1 INTRODUCTION

Diabetes has turned into one of the most common non‐communicable diseases in recent years. According to a World Health Organization report, 422 million people worldwide had diabetes in 2014, which shows a triple increase in the prevalence of this condition compared to 1980 (Roglic, 2016). The global prevalence of diabetes was reported as 8.51% in 2017 (Roglic, 2016). The prevalence of diabetes was reported as 12% in 2011 in Iran. It is estimated that 9.2 million Iranians will have diabetes by 2030 (Esteghamati et al., 2017). One of the solutions for the control of non‐communicable diseases worldwide is primary prevention in the target population. The basis of the primary prevention of non‐communicable diseases is the identification, prevention, and control of their primary risk factors (Peykari et al., 2017). One of the groups at a high risk of developing diabetes is people with pre‐diabetes, which is the mid‐stage between normal glucose levels and the development of type‐II diabetes (Khan et al., 2019).

Meanwhile, in some studies, psychological distresses including long‐term depression, anxiety, and stresses have been proposed as risk factors for diabetes. Psychological factors seem to increase the secretion of cortisol by affecting the sympathetic system and the hypothalamus–pituitary–adrenal axis, which leads to insulin resistance, dyslipidemia, obesity, and type‐II diabetes (Hunter, 2016). In one study, the relationship of psychological distresses with insulin resistance was investigated in middle‐aged Swedish people, which held true for men, but not significantly so for women (Eriksson et al., 2008). A meta‐analysis conducted in 2013 showed a slight but significant relationship between depression and insulin resistance, but the direction and mechanism of this relationship is not entirely clear, and further interventional studies were recommended (Kan et al., 2013). Another study suggested that there is a relationship between higher phobic anxiety and the development of diabetes in women (Farvid et al., 2014). Another study also conducted in Sweden showed that depression has a significant relationship with an increased risk of developing diabetes, and this relationship became stronger as the severity of depression increased. Moreover, irrespective of the severity of depression, severe and moderate anxiety both increased the risk of developing type‐II diabetes (Deleskog et al., 2019).

According to some studies, reduced quality and quantity of sleep were linked to diabetes (Cespedes et al., 2016). A recent study reported a relationship between pre‐diabetes and poor sleep quality (Iyegha et al., 2019). Another study investigating the duration of sleep and fasting glucose disorder in Korean adults showed that a reduced duration of sleep is linked to an increased risk of fasting glucose disorder in men, but not in women (Kim et al., 2016). These studies have all recommended conducting more extensive studies on the relationship of psychological distress and sleep quality with pre‐diabetes and diabetes.

The use of supplements has become commonplace in recent years, and some evidence suggests that vitamin D has a role in the progression of diabetes (Sacerdote et al., 2019). Moreover, the relationship between psychological distresses and vitamin D has been investigated in many studies. A meta‐analytical study showed that vitamin D deficiency is associated with depression (Anglin et al., 2013). In some studies, taking vitamin D supplements led to a reduction in depression and anxiety and improved sleep quality (Ghaderi et al., 2017; Majid et al., 2018). In some other studies, however, this vitamin had no effect on psychological symptoms (Gonzalez et al., 2018; Kerley et al., 2017). Therefore, the existing evidence leads to the hypothesis that perhaps vitamin D can be effective in preventing diabetes directly by boosting insulin secretion and indirectly by reducing psychological problems.

In the meantime, omega‐3 supplements have been proposed as a bioactive anti‐inflammatory agent for delaying the development of type‐II diabetes in pre‐diabetic people (Bahadoran et al., 2013; Hommelberg et al., 2010; Li, 2015). The effects of omega‐3 on psychological symptoms have also been studied, and in some studies, depression, anxiety, and sleep quality improved as a result of taking omega‐3 (Haberka et al., 2013; Jahangard et al., 2018). Meanwhile, in some other studies, omega‐3 was reported ineffective on psychological symptoms and sleep quality (Dean et al., 2011; Taheri et al., 2019).

Only one study confirmed the positive effects of combined vitamin D and omega‐3 supplementation on metabolic and psychological parameters in women with Poly Cystic Ovarian Syndrome (PCOS) (Jamilian et al., 2018). Given these conflicting reports and the lack of a study on the effects of the concurrent consumption of these two supplements in pre‐diabetic people, the present study was conducted to determine the effect of vitamin D and omega‐3 co‐supplementation on psychological distresses in women of reproductive age with pre‐diabetes and hypovitaminosis D.

## 2 METHODS

### 2.1 Study design and participants

The present triple‐blind, placebo‐controlled, factorial clinical trial was conducted over 8 weeks on 168 women aged 15 to 50 years with pre‐diabetes and hypovitaminosis D. The data presented are part of a larger study whose results have been partly published (Rajabi‐Naeeni et al., 2020). This study was approved by the Ethics Committee of Shahid Beheshti University of Medical Sciences and was registered with the Iranian Registry of Clinical Trials as IRCT20100130003226N17. The study was conducted at Shahid Rastravesh Laboratory at Alborz University of Medical Sciences in Karaj, Iran, from March to August 2019, in accordance with the Declaration of Helsinki.

The study inclusion criteria were: (1) being a woman of reproductive age with pre‐diabetes and a serum fasting glucose of 100–125 mg/dl (American Diabetes Association, 2013; Global Report on Diabetes, 2016); (2) serum vitamin D < 32 ng/ml (Spedding et al., 2013); (3) BMI < 30 kg/m^2^; and (4) willingness to take part in the study. The exclusion criteria were: (1) type‐I or II diabetes or other metabolic and underlying diseases; (2) the use of herbal or chemical medications affecting serum glucose and lipids; (3) use of vitamin D and omega‐3 supplements over the last 6 months; (4) use of medications interacting with omega‐3 and vitamin D; (5) pregnancy or breastfeeding; and (6) non‐adherence to the medication use protocols.

Sampling was carried out at Shahid Rastravesh Laboratory in Karaj. All women of reproductive age whose diabetes screening showed a fasting glucose of 100 to 125 mg/dl (American Diabetes Association, 2013; Global Report on Diabetes, 2016) were contacted on the phone. After explaining the study objectives to them, those who met the inclusion criteria and were willing to take part in the study were invited to the laboratory. Those who attended the laboratory and signed the written consent form underwent glucose and serum vitamin D testing, and were included in the study if their test results confirmed a fasting glucose between 100 and 125 mg/dl and serum vitamin D less than 32 ng/ml (Dean et al., 2011; Jahangard et al., 2018). The participants were ensured of the confidentiality of their data and their right to withdraw from the study at any time. Over the course of the intervention, the participants visited Shahid Rastravesh Laboratory three times. In the first visit, blood samples were taken from them and their height and weight were measured to determine their BMI. The three‐day food record was given to the participants and necessary explanations about how to complete this form were provided by a nutritionist. Three days later, in their second visit, they returned their completed 3‐day food record, and if their pre‐diabetes and hypovitaminosis were confirmed by the test results, they completed the Depression, Anxiety, and Stress Scales‐21 (DASS‐21), the Pittsburgh Sleep Quality Index (PSQI), and the short form of the International Physical Activity Questionnaire (IPAQ). They were then randomly assigned to four groups (receiving only placebos, receiving vitamin D, receiving omega‐3, and receiving both supplements). The third visit was at the end of the eighth week, when they completed the DASS‐21, PSQI, IPAQ, and the 3‐day food record again. By the end of the study, the results obtained for each participant were explained to them in private.

### 2.2 Randomization and intervention

The participants were assigned to four groups by stratified random block sampling as follows:

1. The block randomization method was used to ensure the equal distribution of the participants into the four groups. First, blocks of four were considered in 24 different arrangements (ACDB, ACBD, ABDC, ABCD, etc.). Then, the blocks were selected by randomization method and the participants entered each group according to the order of the chosen blocks.
2. To ensure that equal numbers of participants with vitamin D insufficiency and vitamin D deficiency were allocated to all four groups, a stratified randomization was performed based on serum 25(OH)D concentration, that is, less than 20 and between 20 and 32 ng/ml (Płudowski et al., 2013; Spedding et al., 2013). Randomization was carried out by an expert outside the research team. Thus, the researcher, participants, and the statistician had no knowledge of the group allocations (triple‐blind).

The supplement doses were determined based on the results of some similar studies (Jamilian et al., 2017), as follows: 50,000 IU of vitamin D (cholecalciferol) every 2 weeks, and 1 g twice‐daily of omega‐3.

Zahravi Pharmaceutical Company (Tabriz, Iran) was responsible for preparing the vitamin D supplements (IRC:1228055799/GTIN:06260155960213), omega‐3 supplement (IRC:1228058530/GTIN: 06260155920675), and placebos. The shape, size, color, packaging, scent, and taste of the supplements and placebos were totally similar, and the packages were identified with letters A, B, C, and D. The researcher, participants, and statistician had no knowledge of the content of these packages. The participants were assigned to one of the four groups for 8 weeks using the explained randomization method.

Group 1: Omega‐3 placebo twice daily, and vitamin D placebo once every 2 weeks (placebo‐controlled)
Group 2: 1000 mg of omega‐3 twice daily, and vitamin D placebo once every 2 weeks
Group 3: Omega‐3 placebo twice a day, and 50,000 IU of vitamin D once every 2 weeks
Group 4: 1000 mg of omega‐3 twice a day, and 50,000 IU of vitamin D once every 2 weeks

No side effects were reported in previous studies for these doses of the supplements (Baidal et al., 2016; Jamilian et al., 2017). The study subjects were asked not to change their regular food regimen or physical activity over the 8 weeks of the study.

The participants were called on the phone once a week to prevent sample loss. They were also asked about the side effects of these supplements and reminded not to change their food regimen or physical activity. Moreover, their dose and method of daily use of the supplements were assessed. The participants were given a table on the daily use of supplements to ensure their easy adherence to the intervention protocol. They returned this table and the empty packages of the capsules to the researchers at the end of the intervention. A phone number was also given out to the study subjects to contact the research team if necessary.

### 2.3 Measurements

#### 2.3.1 Questionnaires

Physical activity was measured using the IPAQ. This tool expresses walking and moderate and intense physical activity in (MET)‐min per week. IPAQ classifies the intensity of physical activity as low, moderate, and high (Ainsworth et al., 2000).

The average energy received and the micro‐ and macro‐nutrients used were assessed using the 3‐day food record (two week days and one weekend day) and analyzed in Nutritionist IV (First Databank, San Bruno, CA).

The general DASS‐21 contains 21 items in three subscales, including depression, anxiety, and stress, each with seven items, which are scored based on a 4‐point scale (from 0 to 3), and the final score is the sum of the scores of the items in each subscale multiplied by two (Lovibond & Lovibond, 1995). The validity and reliability of DASS‐21 were confirmed by Lovibond and Lovibond (1995) and Henry and Crawford (2005) and also in different studies in Iran (Moradipanah et al., 2009; Sahebi et al., 2005; Samani & Joukar, 2007).

The symptoms of sleep disorder were assessed using Pittsburgh's Sleep Quality Inventory. This questionnaire has nine items, but since item 5 has ten secondary items, the entire questionnaire therefore has 19 items, with scoring based on a 4‐point Likert scale from 0 to 3 points. PSQI has seven dimensions, including subjective sleep quality, sleep latency, sleep duration, habitual sleep efficiency, sleep disturbances, use of sleeping medication, and daytime dysfunction. The total score of sleep quality is the sum of the scores of all the subscales, which is a number between 0 and 21, and scores > 5 suggest abnormal sleep quality (Buysse et al., 1989). The validity and reliability of the original version were confirmed by Dr. Boysse et al. (Buysse et al., 1989) and also in several studies in Iran (Afkham Ebrahimi et al., 2008; Malek et al., 2011).

#### 2.3.2 Anthropometrindices

At the start of the intervention, participants’ height and weight were measured to determine their BMI. Body weight was measured in fasting mode, with no shoes, minimum clothing, and using a digital scale with an accuracy of 0.1 kg (Beurer BF 220, Germany), and height in a standing position, with no shoes, the heel touching the wall, and using a stadiometer with an accuracy of 0.1 cm (Seca, Hamburg, Germany). BMI was determined as weight in kilograms divided by height in meters squared (Aghasi et al., 2018).

#### 2.3.3 Blood samples and biochemical assessments

At the start of the intervention, 10 cc venous blood samples were taken from all the participants after 12 h of fasting. The serum was separated by centrifuging at 3000 rpm for 10 min and kept at −80°C until testing time (Aghasi et al., 2018). The fasting glucose test was carried out using spectrophotometry with Pars Azmoon kits (made in Iran) in an auto‐analyzer (BT3000, Italy). The inter‐assay CVs and intra‐assay CVs of the serum glucose test were less than 7%.

Vitamin D was assessed using the ELISA technique in Stat Fax 4200 (USA) and Monobind CA kit. The inter‐assay CVs and intra‐assay CVs of the vitamin D test were 5% and 6%, respectively.

### 2.4 Sample size calculation

Similar to the results of another study on this subject (Jamilian et al., 2018), after the intervention, the mean and standard deviation of changes in the score of Beck's depression inventory (BDI) were measured as −0.5 and 0.6 in the placebo group and −1.4 and 1.6 in co‐supplementation group. Based on the relevant equation, type‐one error of 5% and test power of 80%, the sample size was determined as 28 per group. Given the factorial type of the study and the use of the related coefficient (Freidlin & Korn, 2017), the size of each group was increased to 36, and to take account of a potential sample loss of 15%, the sample size per group was further increased to 42. The study was thus conducted on 168 people in four groups.

### 2.5 Statistical analysis

The analysis was carried out both as intention‐to‐treat and according to the protocol (Rajabi‐Naeeni et al., 2019). The present report is based on the intention‐to‐treat analysis. The normal distribution of the data was assessed using the Kolmogorov‐Smirnov test. Considering the normality of all the quantitative variables, the results were expressed as mean and standard deviation, and as percentage for the qualitative variables. The one‐way ANOVA was used to determine the difference between the four groups at the start of the study in terms of the scores of depression, anxiety, stress, sleep quality, age, BMI, physical activity, and dietary intakes. Differences between the intervention groups in terms of the qualitative variables at the start of the study were assessed using the chi‐squared test.

The paired *t*‐test was used to compare the mean scores of depression, anxiety, stress, sleep quality, physical activity, and dietary intakes before and after the intervention in each group.

The two‐way ANOVA with repeated measures plus Bonferroni's correction were used to compare the groups and also to assess the interaction of time*treatment (vitamin D/omega‐3) on all the variables between the groups. Data were analyzed in SPSS‐24. All the results were reported with a 95% confidence interval and *p* < .05.

## 3 RESULTS

### 3.1 Subject characteristics

**FIGURE 1:**
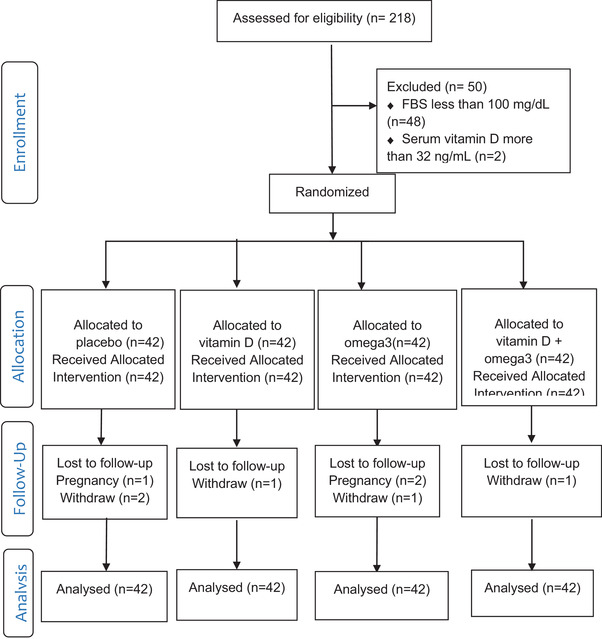
Flow diagram of the study

As shown in Figure 1, the study ended with 160 participants. Three participants were excluded from the study due to getting pregnant and five for personal reasons. The data were analyzed in both the intention‐to‐treat and per‐protocol forms, which produced similar results. The present report is based on the intention‐to‐treat analysis conducted on 168 participants. Participants’ mean age was 40.14 ± 7.06 years. A total of 55.4% of the participants reported diabetes in their first‐degree relatives and 53.6% in their second‐degree relatives.

There were no significant differences between the four groups at the start of the intervention in the mean age, BMI, scores of psychological distress (depression, anxiety, stress, and sleep quality), physical activity, dietary intakes and time spent out door (Table 1).

**TABLE 1:** Baseline characteristics of the participants by group

Characteristic	Placebo group (*n* = 42)	Omega‐3 group (*n* = 42)	Vitamin D group (*n* = 42)	Vitamin D + Omega‐3 group (*n* = 42)	*p*‐Value^a^
Age (years)	41.85 (7.48)	39.78 (6.88)	39.92 (6.04)	39.00 (7.68)	.29
Vitamin D (ng/d)	25.47 (5.83)	23.56 (6.42)	21.43 (8)	22.03 (6.92)	.44
FBS (mg/dl)	104.52 (6.39)	103.87 (5.88)	103.74 (5.11)	103.71 (4.73)	.98
Depression	13.32 (11.38)	12.95 (9.51)	12.37 (10.74)	16.09 (10.42)	.25
Anxiety	10.79 (9.28)	10.71 (8.31)	9.62 (7.82)	13.34 (9.73)	.21
Stress	16.35 (10.61)	18.51 (9.16)	16.83 (12.09)	20.20 (10.41)	.29
Sleep quality	6.32 (3.35)	5.58 (2.97)	6.07 (3.96)	6.37 (2.97)	.74
BMI (kg/m^2^)	27.28 (2.74)	27.17 (2.74)	27.01 (2.91)	27.04 (2.86)	.82
Dietary energy intake (kcal)	1639.0 (601.9)	1645.5 (451.8)	1523 (499.6)	1570.60 (509.8)	.26
Physical activity (MET‐min/week)	698.19 (618.83)	815.34 (778.54)	706.78 (656.76)	808.66 (766.99)	.85
Time spent out door(h/day)	1.7 (1.8)	1.8 (1.2)	1.8 (1.4)	1.8 (1.8)	.88

*Note*: All values are means (SDs).

Abbreviations: BMI, body mass index; FBS, fasting blood sugar.

*p‐Value is significant.(None of data in column P‐ value of table1 is significant. so any “*” is not shown in the table)

^a^Obtain from one‐way analysis of variance.

There was no significant difference within any of the groups in the pre‐ and post‐intervention comparison of physical activity and dietary intakes (micronutrients and macronutrients) and time spent out door.

### 3.2 Effect on DASS‐21 score

Tables 2 and 3 present the effects of using the supplements on psychological symptoms.

**TABLE 2:** Comparison of psychologic distress and vitamin D in each group before and after the intervention

Variables	Time	Group 1 Placebo group (*n* = 42)	Group 2 Omega‐3 group (*n* = 42)	Group 3 Vitamin D group (*n* = 42)	Group 4 Vitamin D + Omega‐3 group (*n* = 42)
Depression	Before	13.32 (11.38)	12.95 (9.51)	12.37 (10.74)	16.09 (10.42)
	After	13.54 (11.27)	10.95 (9.12)	10.25 (10.16)	10.37 (8.69)
	*p‐*Value^a^	0.56	<.001*	<.001*	<.001*
Anxiety	Before	10.79 (9.28)	10.71 (8.31)	9.62 (7.82)	13.34 (9.73)
	After	11.48 (9.38)	8.27 (7.19)	7.79 (7.49)	9.29 (7.75)
	*p*‐Value^a^	0.88	<.001*	<.001*	<.001*
Stress	Before	16.35 (10.61)	18.51 (9.16)	16.83 (12.09)	20.20 (10.41)
	After	16.01 (10.17)	14.66 (9.76)	15.27 (11.27)	13.94 (8.98)
	*p*‐Value^a^	.07	<.001*	<.001*	<.001*
Sleep quality	Before	6.32 (3.35)	5.58 (2.97)	6.07 (3.96)	6.37 (2.97)
	After	6.41 (3.82)	4.85 (3.06)	5.49 (3.14)	4.67 (2.39)
	*p*‐Value^a^	.49	<.001*	<.001*	<.001*
Vitamin D	Before	25.47 (5.83)	23.56 (6.42)	21.43 (8)	22.03 (6.92)
	After	20.97 (6.63)	21.17 (7.40)	29.71 (12.11)	31.71 (11.51)
	*p*‐Value^a^	<.001*	<.001*	<.001*	<.001*

*Notes*: All values are means (SDs).

^a^Obtain from paired samples *t*‐test.

*p‐Value is significant.

**TABLE 3:** Adjusted changes in psychologic distress in each group

	Group 1 Placebo group (*n* = 42)	Group 2 Omega‐3 group (*n* = 42)	Group 3 Vitamin D group (*n* = 42)	Group 4 Vitamin + Omega‐3 group (*n* = 42)	*p*‐Value¥		ηp2¥	
Variables					Time	Time*group interaction	Time	Time*group interaction
Depression	0.21 (6.11)	−1.99 (5.77)	−2.11 (7.05)	−5.71 (5.48) €	<.001*	<.001*	.13	.11
Anxiety	0.69 (3.33)	−2.44 (5.95)	−1.83 (4.57)	−4.05 (5.47) ǁ	<.001*	<.001*	.13	.11
Stress	−0.34 (8.01)	−3.78 (7.89)	−1.63 (7.34)	−6.23 (7.79) €	<.001*	<.001*	.13	.07
Sleep quality	0.09 (2.33)	−0.73 (1.47)	−0.57 (2.28)	−1.7 (1.78) ǁ	<.001*	<.001*	.11	.09

*Notes*: All values are means (SDs).

¥ obtain from two‐way repeated measures analysis of variance after adjusting for covariates.

€ Significant difference with placebo group.

ǁ Significant difference with other three groups.

(¥, ǁ, #) Obtain from two‐way repeated measures analysis of variance with Bonferroni correction.

ηp^2^ (partial eta squared) = 0.14 or more are large effects, 0.06 to 0.14 are medium effects and Less than 0.6 are small effects.

*p‐Value is significant.

A significant group*time interaction was observed for changes in depression, anxiety, and stress scores (*p* < .001). ηp^2^ (partial eta squared) of group*time interaction in depression, anxiety, and stress showed medium effects (Table 3).

By the end of the study, the depression score reduced significantly in the co‐supplementation, omega‐3, and vitamin D groups (*p* < .001) (Table 2). There was a significant difference in changes in depression scores between the co‐supplementation group and the placebo group (*p* < .05) (Table 3).

After the intervention, the anxiety score reduced significantly in all the groups (*p* < .001), except in the placebo group Table 2). A significant difference was observed between the co‐supplementation group and the three other groups in terms of the changes in anxiety score (*p* < .05) (Table 2).

Although a significant reduction was observed in the stress score in the co‐supplementation, omega‐3, and vitamin D groups (*p* < .001) (Table 2), a significant difference in terms of changes in the stress score was observed only between the co‐supplementation and placebo groups (*p* < .05) (Table 3).

### 3.3 Effect on PSQI score

Tables 2 and 3 present the effects of using the supplements on sleep quality.

A significant group*time interaction was observed for changes in the sleep quality score (*p* < .001). ηp^2^ (partial eta squared) of group*time interaction in the sleep quality score showed small effect (Table 3).

At the end of the study, a significant reduction was observed in the sleep quality score in the co‐supplementation, omega‐3, and vitamin D groups (*p* < .001) (Table 2). There were significant differences between the co‐supplementation group and the other three groups in terms of changes in the sleep quality score (*p* < .05) (Table 3).

At the beginning of the intervention, 59.5% of the omega‐3 and vitamin D groups and 47.6% of the placebo and co‐supplementation groups had normal sleep quality, while after the intervention, 64.3% of the omega‐3 and co‐supplementation groups and 61.9% of the vitamin D group achieved normal sleep quality, but normal sleep quality reduced to 45.2% in the placebo group by the end of the study.

After adjusting the co‐variances (age, BMI, physical activity, and nutrient intake), the repeated measures ANOVA showed no changes in any of these findings.

### 3.4 Safety and compliance

Participants’ compliance was 97% over the course of the study. There was no report of side effects due to the use of the supplements during the study.

## 4 DISCUSSION

The present study confirmed the beneficial effects of co‐supplementation with vitamin D and omega‐3 for 8 weeks in women of reproductive age with pre‐diabetes and hypovitaminosis D in improving depression, anxiety, stress, and sleep quality. In some studies, psychological distresses (depression, anxiety, and long‐term stresses) have been proposed as risk factors for diabetes (Hunter, 2016). Given their positive effects on mental health, the concurrent use of these two supplements can be further considered as a measure for preventing type‐II diabetes.

Studies have shown that vitamin D has many potential neuroendocrine mechanisms that affect psychological symptoms:

The active form of vitamin D3 can overcome the blood‐brain barrier and bind to vitamin D receptors in cerebral regions involved in depression, such as the prefrontal cortex and hypothalamus (Marsh et al., 2017).

As a neuroactive steroid, vitamin D3 may affect the gamma aminobutyric acid (GABA) system and play a mood‐regulatory role (Mann et al., 2014).

Vitamin D can increase the expression of genes encoded for tyrosine hydroxylase in the stage of synthesis of the neurotransmitters affecting mood, such as dopamine and norepinephrine (Marsh et al., 2017). Moreover, other studies have reported that vitamin D may improve depression symptoms by regulating the synthesis of serotonin in the brain (Ghaderi et al., 2017).

Vitamin D3 also strengthens the nerve growth factor (NGF) and glial derived neurotrophic factor (GDNF), and GDNF may have a role in depression (Marsh et al., 2017).

Vitamin D3 has a neuroprotective effect through various mechanisms, including reducing Ca2+ concentration in the brain and enhancing the antioxidant defense system (Eyles et al., 2013).

The present findings were similar to those of Ghaderi's study in the vitamin D supplementation group, as a significant improvement was observed in vitamin D, anxiety, depression, and sleep quality in the intervention group in that study. A significant difference was observed between the intervention and placebo groups in vitamin D, psychological symptoms, and sleep quality (Ghaderi et al., 2017). Nonetheless, some studies have reported different results, which may be due to the differences in participants’ age and gender (18), participants’ illnesses (15, 18), and vitamin D dosage (Gonzalez et al., 2018; Kaplan et al., 2015).

Most studies have reported beneficial effects for omega‐3 on psychological distress. In a meta‐analysis of 31 articles published by 2015, Grosso et al. (2016) reported that using omega‐3 reduces the risk of depression. According to various studies, the mechanisms of effect of omega‐3 on physiological symptoms are focused on two main pillars:

Omega‐3, especially DHA and EPA, reduce the synthesis of eicosanoids from arachidonic acid and subsequently cause a drop in pro‐inflammatory cytokines (Wani et al., 2015). Pro‐inflammatory cytokines stimulate the secretion of corticotropin‐releasing hormones (CRH) from the hypothalamus (Kiecolt‐Glaser et al., 2011). CRH leads to the release of pituitary corticotropin, adrenal stimulation, and increased plasma concentration of epinephrine and cortisol from the adrenal gland, which entail other hormonal responses to stress (Barbadoro et al., 2013). Moreover, CRH stimulates the amygdala in the brain, which is a key region for fear and anxiety (Kiecolt‐Glaser et al., 2011). The inflammatory cytokines are also linked to depression and bipolar disorder (Wani et al., 2015).

Omega‐3 has a major role in maintaining the integrity and fluidity of the cell membrane. It thus affects the number and function of receptors, the function of iron channels, and the synthesis of neurotransmitters and neurotrophins (Jahangard et al., 2018). The accumulation of DHA and EPA in synapses is required for the serotonergic and dopaminergic function, and in this way, omega‐3 exerts its psychoactive effects (Haberka et al., 2013).

The findings obtained in the omega‐3 group in this study are similar to those obtained by Kiecolt‐Glaser et al. (2011) which showed no significant difference between the intervention and placebo groups in terms of sleep quality, stress, and depression, although there was a significant difference between the two groups in terms of anxiety score. The results obtained by Sohrabi et al. (2013) on the effects of omega‐3 on anxiety, stress, and depression also agree with the present findings. In contrast, a number of studies reported different results due to the differences in their participants’ age (Taheri et al., 2019), physical (Haberka et al., 2013) or mental (Jahangard et al., 2018) illnesses, and doses of omega‐3 (Grosso et al., 2016).

Furthermore, studies have shown that omega‐3 can cause the activation of vitamin D (Lee et al., 2015). In a study conducted by Jamilian et al. (2018), the effect of the concurrent use of vitamin D and omega‐3 was assessed on metabolic and genetic parameters in women with PCOS. The participants were studied in placebo and intervention groups (daily intake of 2000 mg of omega‐3 and intake of 50,000 IU of vitamin D every 2 weeks). The intervention lasted 12 weeks. Anthropometric indices and vitamin D were measured before and after the intervention. BDI, the General Health Questionnaire‐28 (GHQ‐28), and DASS were also completed in that study. Their results showed a significant increase in serum vitamin D levels in the intervention group after the intervention compared to before. Moreover, a significant difference was observed between the two groups in the serum vitamin D level. A significant reduction was evident at the end of the intervention in the BDI, GHQ‐28, and DASS scores in the co‐supplementation group, and a significant difference was observed between the two groups in the scores of each of these three questionnaires (Jamilian et al., 2018). The findings of the cited study fully agree with the present findings. The reason for this similarity appears to be the similar age and characteristics of the participants (insulin resistance), the supplement doses taken, and the tools used to assess psychological symptoms.

To the researchers’ knowledge, the present study is the first on the effect of the concurrent intake of vitamin D and omega‐3 on psychological distress in pre‐diabetic women. The results suggest that the concurrent intake of these two supplements can be effective in improving serum vitamin D, sleep quality, and the control of psychological symptoms in pre‐diabetic women of reproductive age with hypovitaminosis D.

One of the strengths of the present study was its triple‐blind, factorial, randomized, clinical trial design with an appropriate sample size to achieve a reasonable effect size. Another strength was that the two‐way repeated measures ANOVA with Bonferroni's correction were used to determine the interaction of supplementation with psychological distress and compare the groups. To ensure no change in food intake and physical activity during the intervention, the two questionnaires were used before and after the intervention. A weekly phone call was also made to remind the participants not to make any alterations in these two variables and assess their supplement intake and the incidence of side effects. One study limitation was the short duration of supplementation in the groups, which means that the long‐term effects of taking these supplements in postponing diabetes could not be demonstrated.

In conclusion the concurrent intake of vitamin D and omega‐3 improved depression, anxiety, and sleep quality in women of reproductive age with pre‐diabetes and hypovitaminosis D and can therefore be recommended for improving mental health in this group of women.

## AUTHOR CONTRIBUTIONS

Masoumeh Rajabi‐Naeeni, Mahran Dolatian, Mostafa Qorbani, and Amir Abbas Vaezi conceived and developed the idea for the paper and revised the manuscript. Masoumeh Rajabi‐Naeeni contributed to data collection. Mostafa Qorbani contributed to statistical interpretations. All authors read and approved the final manuscript.

### PEER REVIEW

The peer review history for this article is available at https://publons.com/publon/10.1002/brb3.2342

## Data Availability

The datasets used during the current study are available from the corresponding author on reasonable request.
